# Supplementary material for: Analysis of PPARγ Signaling Activity in Psoriasis
Source: Int J Mol Sci. 2021 Aug 10;22(16):8603. doi: 10.3390/ijms22168603 (PMC8395241; doi:10.3390/ijms22168603)
Supplement: Supplementary file 1 [file ijms-22-08603-s001.zip › Supplemental materials_Analysis of PPARg signaling activity in psoriasis/Pathway models/Models images and html files/Differentiation of psoriatic T cells/6359.html]

IL10


# Protein IL10

|  |  |
| --- | --- |
| URN | urn:agi-llid:3586 |
| Total Entities | 0 |
| Connectivity | 12874 |
| Name | IL10 |
| Description | interleukin 10 |
| Notes | The protein encoded by this gene is a cytokine produced primarily by monocytes and to a lesser extent by lymphocytes. This cytokine has pleiotropic effects in immunoregulation and inflammation. It down-regulates the expression of Th1 cytokines, MHC class II Ags, and costimulatory molecules on macrophages. It also enhances B cell survival, proliferation, and antibody production. This cytokine can block NF-kappa B activity, and is involved in the regulation of the JAK-STAT signaling pathway. Knockout studies in mice suggested the function of this cytokine as an essential immunoregulator in the intestinal tract. Mutations in this gene are associated with an increased susceptibility to HIV-1 infection and rheumatoid arthritis.[provided by RefSeq, May 2011] |
| Primary Cell Localization | Extracellular |
| Class | Ligand |
| ObjectType | Protein |

---

|  |  |
| --- | --- |
| Pathway | Cortisol in Resolving Inflammation |
|  | Dectin-1 (CLEC7A) Signaling |
|  | DC-SIGN (CD209) Signaling |
|  | TLR4 Signaling in Leukocytes |
|  | Treg-Cell Differentiation |
|  | Th2-Cell Differentiation |
|  | AHR Signaling in Treg and Dendritic Cells Function |
|  | AHR Signaling in Th17 Cells Function |
|  | AHR Signaling in Tr1 Cells Function |
|  | MC1R in anti-Inflammatory Signaling |
|  | Keep out Signal: LTF Inhibits Neutrophil Recruitment and Inflammation |
|  | Mast-Cells De Novo Synthesized Mediators via IgE Independent Signaling |
|  | CD16/CD14 Proinflammatory Monocyte Activation |
|  | anti-Inflammatory Function of Macrophage M2 Lineage |
|  | Nociception Expression Targets Signaling |
|  | CCR1 Expression Targets |
|  | BDKRB1/2 -> Interleukins Production |
|  | CNR1/2 -> IL1B/2/4/6/10 Production |
|  | HRH2/4 -> IL6/10 Production |
|  | Leptin -> CD25/IL6/IL10 Production |
|  | P2RY11/13/14 -> IL8/10 Production |
|  | Proteins Involved in Atherosclerosis |
|  | Proteins Involved in Dilated Cardiomyopathy |
|  | Proteins Involved in Arterial Hypertension |
|  | Proteins Involved in Myocardial Ischemia |
|  | Proteins Involved in Myocarditis |
|  | Proteins Involved in Acne Vulgaris |
|  | Genes with Mutations Associated with Atopic Dermatitis |
|  | Acute Phase in Atopic Dermatitis |
|  | Proteins Involved in Atopic Dermatitis |
|  | Atopic Dermatitis |
|  | Proteins Involved in Melanoma |
|  | Proteins Involved in Psoriasis |
|  | Genes with Mutations Associated with Psoriasis |
|  | T-Cells Differentiation Block in Psoriasis |
|  | Proteins with Altered Expression in Psoriasis |
|  | Apoptotic Keratinocytes Clearance Recession in Systemic Lupus Erythematosus |
|  | Dendritic Cell Activation in Systemic Lupus Erythematosis |
|  | Proteins Involved in Systemic Lupus Erythematosus |
|  | Th1-Cell Function in Systemic Lupus Erythematosus |
|  | Th2-Cell Function in Systemic Lupus Erythematosus |
|  | Proteins Involved in Vitiligo |
|  | Genes with Mutations Associated with Vitiligo |
|  | Humoral Immunity in Vitiligo |
|  | Treg-Cell Function in Diabetes Mellitus Type 1 |
|  | Natural Killer T-Cell Roles in Diabetes Mellitus Type 1 |
|  | Proteins Involved in Diabetes Mellitus Type 1 |
|  | Treg-Cell Activation in Diabetes Mellitus |
|  | Proteins Involved in Diabetic Nephropathy |
|  | Proteins Involved in Insulin Resistance |
|  | Proteins Involved in non-Alcoholic Fatty Liver Disease |
|  | Proteins Involved in Myasthenia Gravis |
|  | Proteins Involved in Obesity |
|  | Crohn's Disease |
|  | B-Cell Activation in Crohn's Disease |
|  | Th17-Cell Activation in Crohn's Disease |
|  | Proteins Involved in Inflammatory Bowel Diseases |
|  | Dendritic Cell Dysfunction in Crohn's disease |
|  | Dendritic Cell Function in Ulcerative Colitis |
|  | Nicotine anti-Inflammatory Effect in Ulcerative Colitis |
|  | Proteins Involved in Ulcerative Colitis |
|  | Th2-Cell Function in Ulcerative Colitis |
|  | Diffuse Large-B-Cell Lymphoma |
|  | Diffuse Large-B-Cell Lymphoma ABC Subtype |
|  | Proteins Involved in Diffuse Large-B-Cell Lymphoma |
|  | Hodgkin and Reed-Sternberg Cells in Hodgkin Lymphoma |
|  | Proteins Involved in Hodgkin Lymphoma |
|  | Hodgkin Lymphoma |
|  | Mantle Cell Lymphoma |
|  | Proteins Involved in Multiple Myeloma |
|  | Proteins Involved in Hepatocellular Carcinoma |
|  | Th2-Cell Response in Asthma |
|  | Proteins Involved in Gout |
|  | Proteins Involved in Osteoarthritis |
|  | Proteins Involved in Psoriatic Arthritis |
|  | Proteins Involved in Rheumatoid Arthritis |
|  | B-Cells Function in Systemic Scleroderma |
|  | Th2-Cells Function in Systemic Scleroderma |
|  | Genes with Mutations Associated with Systemic Scleroderma |
|  | Proteins Involved in Systemic Scleroderma |
|  | Proteins with Altered Expression in Systemic Scleroderma |
|  | Proteins Involved in HIV Type 1 Infection |
|  | CD4+ T-Cell Function Decline in HIV |
|  | Proteins Involved in Periodontitis |
|  | Gluten Impact on Neuronal System (Hypothesis) |
|  | Proteins Involved in Osteoporosis |
|  | Proteins Involved in Alzheimer's Disease |
|  | Proteins Involved in Astrocytoma |
|  | Proteins Involved in Glioma |
|  | Proteins Involved in Migraine |
|  | Proteins Involved in Parkinson's Disease |
|  | Proteins Involved in Multiple Sclerosis |
|  | Proteins Involved in Endometriosis |
|  | Proteins Involved in Spontaneous Abortion |
|  | Proteins Involved in Age-Related Macular Degeneration |
|  | IL10/STAT3 Signaling in M2 Macrophage and Retinal Angiogenesis |
|  | Proteins with Altered Expression in Age-Related Macular Degeneration |
|  | Proteins Involved in Otitis Media |
|  | Alveolar Macrophage Negative Regulation Declined in COPD |
|  | Proteins Involved in Chronic Obstructive Pulmonary Disease |
|  | Noradrenaline/Gs Expression Targets |
|  | Dopamine/Gs Expression Targets |
|  | Noreadrenaline/Gq Expression Targets |
|  | Epinephrine/Gs Expression Targets |
|  | Epinephrine/Gq Expression Targets |
|  | Dopamine/Gi Expression Targets |
|  | Elevated Receptors -> Expression Targets in Adipose Tissue |
|  | CD2 Expression Targets |
|  | CD19 Expression Targets |
|  | CD8 -> STAT Expression Targets |
|  | Elevated Receptors -> Expression Targets in Blood vessel |
|  | Elevated Receptors -> Expression Targets in Blood Vessel |
|  | CXCL12 Expression Targets |
|  | CCL3 Expression Targets |
|  | CSF2 -> NF-kB Expression Targets |
|  | CSF2 -> STAT Expression Targets |
|  | Clozapine Induced Granulocytopenia |
|  | EGF -> CTNN Expression Targets |
|  | EGF -> AP-1/ATF Expression Targets |
|  | EGF -> CREB/CREBBP/ELK/SRF/MYC Expression Targets |
|  | EGF -> STAT Expression Targets |
|  | F2 -> AP-1/CREB/ELK/SRF/SP1 Expression Targets |
|  | F2 -> STAT1/NF-kB Expression Targets |
|  | Elevated Receptors -> Expression Targets in Colon |
|  | Elevated Receptors -> Expression Targets in Appendix |
|  | HGF -> STAT Expression Targets |
|  | HGF -> AP-1/CREB/ELK/SRF/MYC Expression Targets |
|  | STAT3 Inhibits Maturation of Dendritic Cells and Facilitates Cancer Progression |
|  | STAT3 Facilitates the Function of Treg Cells and Cancer Progression |
|  | IL12 Antitumor Effect is Blocked in Cancer |
|  | Proteins with Altered Expression in Tumor-Promoting Inflammation |
|  | Treg Cells Promote Immunosuppression in Cancer Immune Escape |
|  | Tumor Infiltrating Macrophages in Cancer Progression and Immune Escape |
|  | Myeloid Derived Suppressor Cells in Cancer Immune Escape |
|  | IDO1 in Cancer Immune Escape |
|  | Proteins with Altered Expression in Cancer Immune Escape |
|  | INHBA/ACVR2/BMPR Expression Targets |
|  | Insulin -> STAT Expression Targets |
|  | Insulin -> CEBPA/CTNNB/FOXA/FOXO Expression Targets |
|  | Insulin -> MEF/MYOD Expression Targets |
|  | Insulin -> ELK/SRF/HIF1A/MYC/SREBF Expression Targets |
|  | IFNLR1-> STAT/NFKB Signaling |
|  | IFNA1/IFNR Expression Targets |
|  | IFNB1/IFNR Expression Targets |
|  | IL10R -> STAT Signaling |
|  | IL7 Expression Targets |
|  | IL15 Expression Targets |
|  | IL10 Expression Targets |
|  | IL2 Expression Targets |
|  | Leptin -> STAT Expression Targets |
|  | Leptin -> ELK/SRF Expression Targets |
|  | LPA Expression Targets |
|  | PAF/Gq -> AP-1/ATF1/CREB/ERK/SRF Expression Targets |
|  | Dronabinol/Anandamide Expression Targets |
|  | PAF/Gq -> NF-kB Expression Targets |
|  | PGE1 Expression Targets |
|  | LDLR -> Expression Targets in Lymphoid System and Blood |
|  | Elevated Receptors -> Expression Targets in Lymph Node |
|  | Elevated Receptors -> Expression Targets in Lymphoid System and Blood |
|  | F2R/F2RL1 -> Expression Targets in Lymphoid System and Blood |
|  | CD Markers -> Expression Targets in Lymphoid System and Blood |
|  | GPCRs Family -> Expression Targets in Lymphoid System and Blood |
|  | TNF Receptors -> Expression Targets in Lymphoid System and Blood |
|  | Immunoglobulin Superfamily -> Expression Targets in Lymphoid System and Blood |
|  | CD40 -> Expression Targets in Thymus |
|  | CD40 -> Expression Targets in Lymph Node |
|  | C5AR1 -> Expression Targets in Lymphoid System and Blood |
|  | IgE Receptors -> Targets in Lymphoid System and Blood |
|  | Chemokine Receptor Family -> Expression Targets in Lymphoid System |
|  | Elevated Receptors -> Expression Targets in Thymus |
|  | NGF -> SMAD3/NF-kB Expression Targets |
|  | NGF -> CREB/CEBPB/MEF2A Expression Targets |
|  | NOTCH Expression Targets |
|  | Adenosine Expression Targets |
|  | POMC Expression Targets |
|  | EDN1 Expression Targets |
|  | AGT -> CREB Expression Targets |
|  | IFNA1/Gq Expression Targets |
|  | AGT -> STAT Expression Targets |
|  | PRL/GHR -> NF/kB/ELK/SRF/MYC Expression Targets |
|  | PDCD1 -> ATF/CREB/CREBBP Expression Targets |
|  | PDCD1 -> STAT Expression Targets |
|  | PDCD1 -> AP-1 Expression Targets |
|  | Collagen -> NF-kB Expression Targets |
|  | PAF Expression Targets |
|  | TCR -> CREB/CREBBP/ATF Expression Targets |
|  | TCR -> NF-kB Expression Targets |
|  | TCR -> NFAT Expression Targets |
|  | TCR -> STAT Expression Targets |
|  | TGFB2-TGFBR1 Expression Targets |
|  | TGFB1-TGFBR1 Expression Targets |
|  | TGFB1-ACVRL1 Expression Targets |
|  | FASLG Expression Targets |
|  | CD40LG -> STAT Expression Targets |
|  | CD40LG -> NF-kB/ELK/SRF -> CREB/NFATC Expression Targets |
|  | TLR3 Expression Targets |
|  | TLR5 Expression Targets |
|  | TLR7 Expression Targets |
|  | TLR1 -> 2/6 Expression Targets |
|  | TLR4 -> NF-kB/IRF Expression Targets |
|  | TLR9 Expression Targets |
|  | PPAR Psoriasis |
|  | prarg neg,ukn expres targets, ps-positive |
|  | PPARG negative targets |
|  | pparg expr target\_regulators |
|  | Neighbors of disease exacerbation |
|  | Neighbors of tumor progression |
|  | Neighbors of cancer progression |
|  | Neighbors of inflammatory status |
|  | Neighbors of overall survival |
|  | Neighbors of disease severity |
|  | Neighbors of tumor size |
|  | Neighbors of lesion size |
|  | Neighbors of mortality |
|  | Neighbors of survival rate |
|  | Neighbors of infarct size |
|  | Neighbors of survival time |
|  | Neighbors of functional recovery |
|  | Neighbors of insulin sensitivity |
|  | Neighbors of bacterial load |
|  | Neighbors of cancer survival |
|  | Neighbors of graft survival |
|  | Neighbors of therapeutic efficacy |
|  | Neighbors of clinical stage |
|  | Neighbors of lethality |
|  | Neighbors of tumor regression |
|  | Neighbors of recurrence-free survival |
|  | Neighbors of disease-free survival |
|  | Neighbors of tumor response |
|  | Neighbors of macrophage count |
|  | Neighbors of long-term survival |
|  | Neighbors of treatment outcome |
|  | Neighbors of bone mass |
|  | Neighbors of body weight |
|  | Neighbors of body weight gain |
|  | Neighbors of cardiovascular risk |
|  | Neighbors of tumor recurrence |
|  | Neighbors of tumor promotion |
|  | Neighbors of neutrophil count |
|  | Neighbors of glucose tolerance |
|  | Neighbors of morbidity |
|  | Neighbors of fibrosis progression |
|  | Neighbors of mortality rate |
|  | Neighbors of fat mass |
|  | Neighbors of exhaustion |
|  | Neighbors of bone mineral density |
|  | Neighbors of adverse outcome |
|  | Neighbors of lesion area |
|  | Neighbors of cell density |
|  | Neighbors of cancer-specific survival |
|  | Neighbors of progression-free survival |
|  | Neighbors of apoptosis rate |
|  | Neighbors of early mortality |
|  | Neighbors of viral load |
|  | Neighbors of blood pressure |
|  | Neighbors of leukocyte count |
|  | Neighbors of tumor grade |
|  | Neighbors of vascular reactivity |
|  | Neighbors of vascular leakage |
|  | Neighbors of body mass index |
|  | Neighbors of vascular density |
|  | Neighbors of muscle mass |
|  | Neighbors of cancer risk |
|  | Neighbors of cancer incidence |
|  | Neighbors of tumor aggressiveness |
|  | Neighbors of systolic blood pressure |
|  | Neighbors of tumor resistance |
|  | Neighbors of blood glucose |
|  | Neighbors of ejection fraction |
|  | Neighbors of median survival |
|  | Neighbors of renal hypertrophy |
|  | Neighbors of osteoclast number |
|  | Neighbors of T-cell count |
|  | Neighbors of antimetastatic activity |
|  | Neighbors of energy expenditure |
|  | Neighbors of glucose level |
|  | Neighbors of vascular tone |
|  | Neighbors of left ventricular ejection fraction |
|  | Neighbors of breast cancer risk |
|  | Neighbors of pregnancy outcome |
|  | Neighbors of functional status |
|  | Neighbors of gastric cancer risk |
|  | Neighbors of neurological outcome |
|  | Neighbors of infarct area |
|  | Neighbors of fertility |
|  | Neighbors of recurrence rate |
|  | Neighbors of pathological response |
|  | Neighbors of lymphocyte count |
|  | Neighbors of monocyte count |
|  | Neighbors of skin thickness |
|  | Neighbors of metabolic response |
|  | Neighbors of comorbidity |
|  | Neighbors of carotid intima-media thickness |
|  | Neighbors of disease exacerbation |
|  | Neighbors of tumor progression |
|  | Neighbors of cancer progression |
|  | Neighbors of inflammatory status |
|  | Neighbors of overall survival |
|  | Neighbors of disease severity |
|  | Neighbors of tumor size |
|  | Neighbors of lesion size |
|  | Neighbors of mortality |
|  | Neighbors of survival rate |
|  | Neighbors of infarct size |
|  | Neighbors of survival time |
|  | Neighbors of functional recovery |
|  | Neighbors of insulin sensitivity |
|  | Neighbors of bacterial load |
|  | Neighbors of cancer survival |
|  | Neighbors of graft survival |
|  | Neighbors of therapeutic efficacy |
|  | Neighbors of clinical stage |
|  | Neighbors of lethality |
|  | Neighbors of tumor regression |
|  | Neighbors of recurrence-free survival |
|  | Neighbors of disease-free survival |
|  | Neighbors of tumor response |
|  | Neighbors of macrophage count |
|  | Neighbors of long-term survival |
|  | Neighbors of treatment outcome |
|  | Neighbors of bone mass |
|  | Neighbors of body weight |
|  | Neighbors of body weight gain |
|  | Neighbors of cardiovascular risk |
|  | Neighbors of tumor recurrence |
|  | Neighbors of tumor promotion |
|  | Neighbors of neutrophil count |
|  | Neighbors of glucose tolerance |
|  | Neighbors of morbidity |
|  | Neighbors of fibrosis progression |
|  | Neighbors of mortality rate |
|  | Neighbors of fat mass |
|  | Neighbors of exhaustion |
|  | Neighbors of bone mineral density |
|  | Neighbors of adverse outcome |
|  | Neighbors of lesion area |
|  | Neighbors of cell density |
|  | Neighbors of cancer-specific survival |
|  | Neighbors of progression-free survival |
|  | Neighbors of apoptosis rate |
|  | Neighbors of early mortality |
|  | Neighbors of viral load |
|  | Neighbors of blood pressure |
|  | Neighbors of leukocyte count |
|  | Neighbors of tumor grade |
|  | Neighbors of vascular reactivity |
|  | Neighbors of vascular leakage |
|  | Neighbors of body mass index |
|  | Neighbors of vascular density |
|  | Neighbors of muscle mass |
|  | Neighbors of cancer risk |
|  | Neighbors of cancer incidence |
|  | Neighbors of tumor aggressiveness |
|  | Neighbors of systolic blood pressure |
|  | Neighbors of tumor resistance |
|  | Neighbors of blood glucose |
|  | Neighbors of ejection fraction |
|  | Neighbors of median survival |
|  | Neighbors of renal hypertrophy |
|  | Neighbors of osteoclast number |
|  | Neighbors of T-cell count |
|  | Neighbors of antimetastatic activity |
|  | Neighbors of energy expenditure |
|  | Neighbors of glucose level |
|  | Neighbors of vascular tone |
|  | Neighbors of left ventricular ejection fraction |
|  | Neighbors of breast cancer risk |
|  | Neighbors of pregnancy outcome |
|  | Neighbors of functional status |
|  | Neighbors of gastric cancer risk |
|  | Neighbors of neurological outcome |
|  | Neighbors of infarct area |
|  | Neighbors of fertility |
|  | Neighbors of recurrence rate |
|  | Neighbors of pathological response |
|  | Neighbors of lymphocyte count |
|  | Neighbors of monocyte count |
|  | Neighbors of skin thickness |
|  | Neighbors of metabolic response |
|  | Neighbors of comorbidity |
|  | Neighbors of carotid intima-media thickness |
|  | Neighbors of cell development |
|  | Neighbors of immune response |
|  | Neighbors of cell population |
|  | Neighbors of T-cell development |
|  | Neighbors of inflammatory response |
|  | Neighbors of wound healing |
|  | Neighbors of angiogenesis |
|  | Neighbors of innate immune response |
|  | Neighbors of cell function |
|  | Neighbors of macrophage activation |
|  | Neighbors of cell formation |
|  | Neighbors of adaptive immune response |
|  | Neighbors of osteoclast development |
|  | Neighbors of epithelial to mesenchymal transition |
|  | Neighbors of immunity |
|  | Neighbors of cell proliferative response |
|  | Neighbors of chemotaxis |
|  | Neighbors of dendritic cell differentiation |
|  | Neighbors of cell survival |
|  | Neighbors of osteoclast differentiation |
|  | Neighbors of cell phenotype |
|  | Neighbors of T-cell proliferation |
|  | Neighbors of tissue remodeling |
|  | Neighbors of cell adhesion |
|  | Neighbors of T-cell activation |
|  | Neighbors of endothelial cell proliferation |
|  | Neighbors of fibrogenesis |
|  | Neighbors of tumor growth |
|  | Neighbors of dendritic cell development |
|  | Neighbors of tissue repair |
|  | Neighbors of cell count |
|  | Neighbors of cell infiltration |
|  | Neighbors of cell interaction |
|  | Neighbors of SMC proliferation |
|  | Neighbors of lumen formation |
|  | Neighbors of endothelial cell migration |
|  | Neighbors of adipogenesis |
|  | Neighbors of lipid storage |
|  | Neighbors of cell damage |
|  | Neighbors of pregnancy |
|  | Neighbors of monocyte differentiation |
|  | Neighbors of aging |
|  | Neighbors of cell motility |
|  | Neighbors of bone resorption |
|  | Neighbors of monocyte migration |
|  | Neighbors of fibroblast proliferation |
|  | Neighbors of adipocyte differentiation |
|  | Neighbors of macrophage migration |
|  | Neighbors of microglial activation |
|  | Neighbors of neutrophil recruitment |
|  | Neighbors of regeneration |
|  | Neighbors of cell migration |
|  | Neighbors of cancer growth |
|  | Neighbors of stem cell proliferation |
|  | Neighbors of smooth muscle cell migration |
|  | Neighbors of hepatic regeneration |
|  | Neighbors of transendothelial migration |
|  | Neighbors of phagocytosis |
|  | Neighbors of immune system activation |
|  | Neighbors of leukocyte migration |
|  | Neighbors of leukocyte recruitment |
|  | Neighbors of osteoclast formation |
|  | Neighbors of monocyte recruitment |
|  | Neighbors of cell invasion |
|  | Neighbors of heart function |
|  | Neighbors of hemopoiesis |
|  | Neighbors of bone remodeling |
|  | Neighbors of ossification |
|  | Neighbors of T-cell function |
|  | Neighbors of immune system function |
|  | Neighbors of lung development |
|  | Neighbors of autophagy |
|  | Neighbors of monocyte adhesion |
|  | Neighbors of transcription activation |
|  | Neighbors of senescence |
|  | Neighbors of T-cell response |
|  | Neighbors of tumor immunity |
|  | Neighbors of hepatocyte proliferation |
|  | Neighbors of cell transdifferentiation |
|  | Neighbors of neuroprotection |
|  | Neighbors of stem cell differentiation |
|  | Neighbors of endothelial cell function |
|  | Neighbors of neuronal death |
|  | Neighbors of cell growth |
|  | Neighbors of ROS generation |
|  | Neighbors of epithelial cell proliferation |
|  | Neighbors of first trimester pregnancy |
|  | Neighbors of cancer cell growth |
|  | Neighbors of sensitization |
|  | Neighbors of hemato-encephalic barrier |
|  | Neighbors of macrophage function |
|  | Neighbors of cellular immune response |
|  | Neighbors of cellular senescence |
|  | Neighbors of colony formation |
|  | Neighbors of macrophage differentiation |
|  | Neighbors of cell differentiation |
|  | Neighbors of keratinocyte proliferation |
|  | Neighbors of stem cell migration |
|  | Neighbors of life span |
|  | Neighbors of cell homeostasis |
|  | Neighbors of psoriasis |
|  | Neighbors of inflammatory disease |
|  | Neighbors of rheumatoid arthritis |
|  | Neighbors of atherosclerosis |
|  | Neighbors of fibrosis |
|  | Neighbors of colitis |
|  | Neighbors of arthritis |
|  | Neighbors of inflammatory bowel disease |
|  | Neighbors of injury |
|  | Neighbors of atherogenesis |
|  | Neighbors of vascular remodeling |
|  | Neighbors of metastasis |
|  | Neighbors of carcinogenesis |
|  | Neighbors of multiple sclerosis |
|  | Neighbors of chronic inflammation |
|  | Neighbors of inflammation |
|  | Neighbors of tumor microenvironment |
|  | Neighbors of pathological angiogenesis |
|  | Neighbors of obesity |
|  | Neighbors of vascular disease |
|  | Neighbors of diabetes mellitus |
|  | Neighbors of asthma |
|  | Neighbors of pneumonia |
|  | Neighbors of hyperplasia |
|  | Neighbors of osteoarthritis |
|  | Neighbors of liver fibrosis |
|  | Neighbors of chronic obstructive pulmonary disease |
|  | Neighbors of myocardial infarction |
|  | Neighbors of leukocyte infiltration |
|  | Neighbors of reperfusion injury |
|  | Neighbors of airway inflammation |
|  | Neighbors of hepatitis |
|  | Neighbors of insulin resistance |
|  | Neighbors of infection |
|  | Neighbors of vascular injury |
|  | Neighbors of renal injury |
|  | Neighbors of preeclampsia |
|  | Neighbors of macrophage infiltration |
|  | Neighbors of liver injury |
|  | Neighbors of neuroinflammation |
|  | Neighbors of sepsis |
|  | Neighbors of death |
|  | Neighbors of type 1 diabetes |
|  | Neighbors of encephalomyelitis |
|  | Neighbors of autoimmune disease |
|  | Neighbors of ischemic stroke |
|  | Neighbors of dermatitis |
|  | Neighbors of neutrophil infiltration |
|  | Neighbors of heart disease |
|  | Neighbors of diabetic nephropathy |
|  | Neighbors of lung injury |
|  | Neighbors of immunopathology |
|  | Neighbors of systemic sclerosis |
|  | Neighbors of hepatocellular carcinoma |
|  | Neighbors of autoimmunity |
|  | Neighbors of acute lung injury |
|  | Neighbors of gastric cancer |
|  | Neighbors of atherosclerotic plaque |
|  | Neighbors of experimental autoimmune encephalomyelitis |
|  | Neighbors of colorectal cancer |
|  | Neighbors of cardiac remodeling |
|  | Neighbors of breast cancer |
|  | Neighbors of ischemia |
|  | Neighbors of systemic lupus erythematosus |
|  | Neighbors of melanoma |
|  | Neighbors of collagen-induced arthritis |
|  | Neighbors of cartilage degeneration |
|  | Neighbors of interstitial fibrosis |
|  | Neighbors of neoplasm invasion |
|  | Neighbors of kidney disease |
|  | Neighbors of heart failure |
|  | Neighbors of lung disease |
|  | Neighbors of pulmonary fibrosis |
|  | Neighbors of cardiovascular disease |
|  | Neighbors of stroke |
|  | Neighbors of cancer |
|  | Neighbors of cerebral ischemia |
|  | Neighbors of type 2 diabetes |
|  | Neighbors of neoplasm |
|  | Neighbors of endometriosis |
|  | Neighbors of neointima |
|  | Neighbors of vasculitis |
|  | Neighbors of atrophy |
|  | Neighbors of diet-induced obesity |
|  | Neighbors of respiratory hypersensitivity |
|  | Neighbors of myocardial fibrosis |
|  | Neighbors of synovitis |
|  | Neighbors of coronary artery disease |
|  | Neighbors of rupture |
|  | Neighbors of endothelial cell dysfunction |
|  | Neighbors of liver disease |
|  | Neighbors of prostate cancer |
|  | Neighbors of hypertrophy |
|  | Neighbors of osteolysis |
|  | Neighbors of ovarian cancer |
|  | Neighbors of pancreatic cancer |
|  | Neighbors of hypertension |
|  | Neighbors of intimal hyperplasia |
|  | Neighbors of allograft rejection |
|  | Neighbors of proteinuria |
|  | Neighbors of epithelial cell |
|  | Neighbors of keratinocyte |
|  | Neighbors of vascular smooth muscle cell |
|  | Neighbors of umbilical vein endothelial cell |
|  | Neighbors of monocyte |
|  | Neighbors of endometrial stromal cell |
|  | Neighbors of alveolar macrophage |
|  | Neighbors of synoviocyte |
|  | Neighbors of macrophage |
|  | Neighbors of adipocyte |
|  | Neighbors of activated monocyte |
|  | Neighbors of fibroblast |
|  | Neighbors of airway epithelial cell |
|  | Neighbors of PBMC |
|  | Neighbors of stromal cell |
|  | Neighbors of airway smooth muscle cell |
|  | Neighbors of osteoblast |
|  | Neighbors of pneumocyte |
|  | Neighbors of endometrium cell |
|  | Neighbors of microglia |
|  | Neighbors of trophoblast |
|  | Neighbors of intestine epithelium cell |
|  | Neighbors of mononuclear cell |
|  | Neighbors of hepatic stellate cell |
|  | Neighbors of hepatocyte |
|  | Neighbors of decidual cell |
|  | Neighbors of kidney mesangial cell |
|  | Neighbors of endothelial cell |
|  | Neighbors of dendritic cell |
|  | Neighbors of Kuppfer cell |
|  | Neighbors of kidney tubule cell |
|  | Neighbors of inflammatory cell |
|  | Neighbors of bone marrow macrophage |
|  | Neighbors of peritoneal macrophage |
|  | Neighbors of osteoclast |
|  | Neighbors of neutrophil |
|  | Neighbors of smooth muscle myocyte |
|  | Neighbors of astrocyte |
|  | Neighbors of bone marrow derived dendritic cell |
|  | Neighbors of activated T-cell |
|  | Neighbors of mesothelial cell |
|  | Neighbors of mononuclear phagocyte |
|  | Neighbors of immunocompetent cell |
|  | Neighbors of granulocyte |
|  | Neighbors of cardiac myocyte |
|  | Neighbors of cancer-associated fibroblast |
|  | Neighbors of myeloid cell |
|  | Neighbors of mesenchymal stroma cell |
|  | Neighbors of cytotrophoblast |
|  | Neighbors of stem cell |
|  | Neighbors of tumor infiltrating macrophage |
|  | Neighbors of sinusoidal cell |
|  | Neighbors of B-cell |
|  | Neighbors of leukocyte |
|  | Neighbors of chondrocyte |
|  | Neighbors of insulin-secreting cell |
|  | Neighbors of mesenchymal cell |
|  | Neighbors of T-cell |
|  | Neighbors of M1 macrophage |
|  | Neighbors of pancreatic stellate cell |
|  | Neighbors of mast cell |
|  | Neighbors of bone marrow stromal cell |
|  | Neighbors of spleen cell |
|  | Neighbors of brain capillary endothelial cell |
|  | Neighbors of neuroglia |
|  | Neighbors of thyroid epithelial cell |
|  | Neighbors of hematopoietic cell |
|  | Neighbors of adipose macrophage |
|  | Neighbors of conventional dendritic cell |
|  | Neighbors of pericyte |
|  | Neighbors of antigen-presenting cell |
|  | Neighbors of epidermis cell |
|  | Neighbors of bone marrow derived mast cell |
|  | Neighbors of phagocyte |
|  | Neighbors of syncytiotrophoblast |
|  | Neighbors of lymphocyte |
|  | Neighbors of Langerhans cell |
|  | Neighbors of decidual natural killer cell |
|  | Neighbors of brain cell |
|  | Neighbors of M2 macrophage |
|  | Neighbors of peritoneum cell |
|  | Neighbors of lymphoid cell |
|  | Neighbors of retina cell |
|  | Neighbors of blood cell |
|  | Neighbors of skin cell |
|  | Neighbors of luteal cell |
|  | Neighbors of peripheral lymphocyte |
|  | Neighbors of activated B-cell |
|  | Neighbors of lymph node cell |
|  | Neighbors of lung |
|  | Neighbors of liver |
|  | Neighbors of kidney |
|  | Neighbors of skin |
|  | Neighbors of intestine |
|  | Neighbors of lymph node |
|  | Neighbors of colon |
|  | Neighbors of airway |
|  | Neighbors of blood vessel |
|  | Neighbors of heart |
|  | Neighbors of aorta |
|  | Neighbors of gastrointestinal tract |
|  | Neighbors of microvessel |
|  | Neighbors of bone |
|  | Neighbors of placenta |
|  | Neighbors of carotid artery |
|  | Neighbors of synovial membrane |
|  | Neighbors of uterus |
|  | Neighbors of esophagus |
|  | Neighbors of periodontium |
|  | Neighbors of retina |
|  | Neighbors of brain |
|  | Neighbors of blood vessel wall |
|  | Neighbors of stomach |
|  | Neighbors of islets of Langerhans |
|  | Neighbors of joint |
|  | Neighbors of cardiovascular system |
|  | Neighbors of pancreas |
|  | Neighbors of prostate |
|  | Neighbors of central nervous system |
|  | Neighbors of kidney glomerulus |
|  | Neighbors of colorectal region |
|  | Neighbors of decidua |
|  | Neighbors of amnion |
|  | Neighbors of immune system |
|  | Neighbors of spleen |
|  | Neighbors of peritoneum |
|  | Neighbors of allantois |
|  | Neighbors of alveolus |
|  | Neighbors of coronary artery |
|  | Neighbors of uterine cervix |
|  | Neighbors of vein |
|  | Neighbors of villus |
|  | Neighbors of bronchus |
|  | Neighbors of glands |
|  | Neighbors of nose |
|  | Neighbors of eye |
|  | Neighbors of cornea |
|  | Neighbors of hippocampus |
|  | Neighbors of lymph node of mesentery |
|  | Neighbors of mammary gland |
|  | Neighbors of ankle joint |
|  | Neighbors of vitreous body |
|  | Neighbors of thymus gland |
|  | Neighbors of cerebral cortex |
|  | Neighbors of myelin sheath |
|  | Neighbors of bladder |
|  | Neighbors of draining lymph node |
|  | Neighbors of nervous system |
|  | Neighbors of mesentery |
|  | Neighbors of nasopharynx |
|  | Neighbors of cervical lymph node group |
|  | Neighbors of periodontal ligament |
|  | Neighbors of salivary gland |
|  | Neighbors of corpus luteum of ovary |
|  | Neighbors of sympathetic nervous system |
|  | Neighbors of ileum |
|  | Neighbors of endocrine system |
|  | Neighbors of small intestine |
|  | Neighbors of aggregated lymphoid follicle of small intestine |
|  | Neighbors of mediastinal lymph node group |
|  | Neighbors of reproductive system |
|  | Neighbors of Fallopian tube |
|  | Neighbors of hypothalamus |
|  | Neighbors of choroid plexus |
|  | Neighbors of NF-kB family |
|  | Neighbors of oxidized LDL |
|  | Neighbors of JNK |
|  | Neighbors of IL1 family |
|  | Neighbors of PPAR |
|  | Neighbors of Jun/Fos |
|  | Neighbors of NFKBI |
|  | Neighbors of PKC |
|  | Neighbors of PI3K |
|  | Neighbors of STAT family |
|  | Neighbors of mitogen-activated protein kinase |
|  | Neighbors of IFNAR ligand |
|  | Neighbors of transforming growth factor |
|  | Neighbors of TLR |
|  | Neighbors of cytokine |
|  | Neighbors of interferon |
|  | Neighbors of interleukin |
|  | Neighbors of JAK |
|  | Neighbors of MEK1/2 |
|  | Neighbors of PDGF |
|  | Neighbors of ERK1/2 |
|  | Neighbors of growth factor |
|  | Neighbors of PKA |
|  | Neighbors of ovalbumin |
|  | Neighbors of LDL |
|  | Neighbors of NF-AT family |
|  | Neighbors of beta adrenoceptor |
|  | Neighbors of chorionic gonadotropin |
|  | Neighbors of IKBK |
|  | Neighbors of adipokine |
|  | Neighbors of protein tyrosine kinase |
|  | Neighbors of IgG |
|  | Neighbors of SMAD subfamily |
|  | Neighbors of AMPK |
|  | Neighbors of LXR |
|  | Neighbors of HDL |
|  | Neighbors of p38 |
|  | Neighbors of inflammatory cytokine |
|  | Neighbors of IL23 |
|  | Neighbors of estrogen receptor |
|  | Neighbors of T-cell receptor |
|  | Neighbors of histone deacetylase |
|  | Neighbors of IL1R |
|  | Neighbors of HIF-1 |
|  | Neighbors of GPCR |
|  | Neighbors of IL12 |
|  | Neighbors of Notch |
|  | Neighbors of prostaglandin-endoperoxide synthase |
|  | Neighbors of heat shock protein 90 |
|  | Neighbors of lectin |
|  | Neighbors of fibrinogen |
|  | Neighbors of interleukin-35 |
|  | Neighbors of endothelin |
|  | Neighbors of NOS |
|  | Neighbors of superoxide dismutase |
|  | Neighbors of proteasome endopeptidase complex |
|  | Neighbors of class nuclear receptor with C4 zinc fingers |
|  | Neighbors of non-coding RNA |
|  | Neighbors of histone H3 |
|  | Neighbors of STAT5 |
|  | Neighbors of vitronectin receptor (integrin) |
|  | Neighbors of PP2A |
|  | Neighbors of SAPK |
|  | Neighbors of WNT |
|  | Neighbors of gonadotropins |
|  | Neighbors of PP2B |
|  | Neighbors of AHR subfamily |
|  | Neighbors of ITG |
|  | Neighbors of endostatins |
|  | Neighbors of CD3 complex |
|  | Neighbors of TLR1/2 heterodimer |
|  | Neighbors of TORC1 |
|  | Neighbors of angiotensin receptor |
|  | Neighbors of RA receptor |
|  | Neighbors of histone |
|  | Neighbors of Rho kinase |
|  | Neighbors of neuropeptides |
|  | Neighbors of pancreatic elastase |
|  | Neighbors of histone acetyltransferase |
|  | Neighbors of adrenoceptor |
|  | Neighbors of phosphodiesterase |
|  | Model of PPARG signaling in psoriasis |
|  | dsRNA induces innate immunity and INFs in alveolar cells |
|  | IFN signaling in alveolar cells |
|  | Dendritic Cell Activation in COVID19 |
|  | Overview. Diseases of the skin (chapter 11) |
|  | Overview. Diseases of the digestive system (chapter 10) |
|  | Overview. Diseases of the ear (chapter 7) |
|  | 2\_Acute phase initiation in atopic dermatitis |
|  | 1\_Differentiation of psoriatic T cells |
|  | 2\_Intestine epithelial cells drive immunological responses in ulcerative colitis |
|  | 4\_1\_Polymorphisms associated with inflammatory bowel diseases |
|  | 3\_Impairment of iron release from splenic macrophages |
|  | 2\_Defective tolerance of autoreactive T-cell in T1D |
|  | drug-target pairs direct regulation |
|  | drug-target moltransport |
|  | New Combined Pathway |
|  | Differentiation of psoriatic T cells |

---

|  |  |
| --- | --- |
| Group | Genes with Mutations Associated with Atopic Dermatitis |
|  | Genes with Mutations Associated with Psoriasis |
|  | Genes Associated with Systemic Lupus Erythematosus |
|  | Genes with Mutations Associated with Vitiligo |
|  | Genes with Mutations Associated with Inflammatory Bowel Diseases |
|  | Genes with Mutations Associated with Systemic Scleroderma |
|  | Genes with Mutations Associated with Multiple Sclerosis |
|  | Genes with Mutations Associated with Endometriosis |

---

|  |  |
| --- | --- |
| Source | curated |
|  | GO component |

---

|  |  |
| --- | --- |
| MedScan ID | 3586 |

---

|  |  |
| --- | --- |
| LocusLink ID | 3586 |
|  | 16153 |
|  | 25325 |
|  | 281246 |
|  | 553957 |

---

|  |  |
| --- | --- |
| Alias | IL10R ligands |
|  | IL10 |
|  | IL10\_HUMAN |
|  | IL10R ligand |
|  | IL10 receptor ligand |
|  | IL10A |
|  | CSIF |
|  | DRG-0229 |
|  | interleukin IL10 |
|  | AL513351.1 |
|  | interleukin IL10A |
|  | Cytokine synthesis inhibitory factor |
|  | OTTMUSP00000017964 |
|  | rhuIL-10 |
|  | LOC113640 |
|  | RP11-262N9.1 |
|  | interleukin 10 |
|  | MGC126451 |
|  | T-cell growth inhibitory factor |
|  | interleukin IL10X |
|  | GVHDS |
|  | interleukin-10 receptor ligand |
|  | MGC126450 |
|  | IL10X |
|  | T-cell growth inhibitory factor (TGIF) |
|  | interleukin IL10\_HUMAN |
|  | TGIF |
|  | IL-10 |
|  | interleukin-10 |
|  | SCH 52000 |
|  | Cytokine synthesis inhibitory factor |
|  | chromosome |
|  | scaffold |

---

|  |  |
| --- | --- |
| GO ID | 0005125 |
|  | 0008083 |
|  | 0005141 |
|  | 0030183 |
|  | 0042100 |
|  | 0007568 |
|  | 0060670 |
|  | 0071392 |
|  | 0035729 |
|  | 0071222 |
|  | 0019221 |
|  | 0007253 |
|  | 0042742 |
|  | 0042832 |
|  | 0072577 |
|  | 0030097 |
|  | 0006954 |
|  | 0035722 |
|  | 0030595 |
|  | 0097421 |
|  | 0030889 |
|  | 0045347 |
|  | 0042130 |
|  | 0043066 |
|  | 0010507 |
|  | 0008285 |
|  | 0071650 |
|  | 0002875 |
|  | 0060302 |
|  | 0001818 |
|  | 0002740 |
|  | 2000352 |
|  | 0044130 |
|  | 0034115 |
|  | 1903208 |
|  | 0050728 |
|  | 0045355 |
|  | 0032689 |
|  | 0032692 |
|  | 0032695 |
|  | 0032701 |
|  | 0032715 |
|  | 0032717 |
|  | 0051045 |
|  | 0045930 |
|  | 0030886 |
|  | 0045019 |
|  | 1904057 |
|  | 0042536 |
|  | 0032720 |
|  | 1904706 |
|  | 0002904 |
|  | 0051091 |
|  | 0046427 |
|  | 0045348 |
|  | 0045787 |
|  | 0050715 |
|  | 0001938 |
|  | 0034116 |
|  | 0043032 |
|  | 1902895 |
|  | 1903672 |
|  | 0045893 |
|  | 1904707 |
|  | 0032800 |
|  | 1903659 |
|  | 0010468 |
|  | 0045191 |
|  | 1903034 |
|  | 0050807 |
|  | 0014823 |
|  | 0034465 |
|  | 0051384 |
|  | 0014854 |
|  | 0032868 |
|  | 0002237 |
|  | 0042092 |
|  | 0005576 |
|  | 0005615 |
|  | 0006955 |
|  | 0045944 |
|  | 0051930 |
|  | 0042493 |
|  | 0032496 |
|  | 0010033 |
|  | 0005622 |
|  | 0006916 |
|  | 0007267 |
|  | 0005737 |
|  | 0043193 |
|  | 0006950 |
|  | 0005515 |
|  | 0007260 |
|  | 0045077 |
|  | 0030236 |
|  | 0042095 |
|  | 0042221 |

---

|  |  |
| --- | --- |
| KEGG ID | hsa:3586 |
|  | mmu:16153 |
|  | rno:25325 |

---

|  |  |
| --- | --- |
| MedLine Reference | 10975994 |
|  | 11244051 |
|  | 11777537 |
|  | 11900340 |
|  | 11943322 |
|  | 12020953 |
|  | 12117955 |
|  | 12162438 |
|  | 12405978 |
|  | 12547408 |
|  | 12565791 |
|  | 12595497 |
|  | 12692173 |
|  | 12707357 |
|  | 12765335 |
|  | 1280414 |
|  | 14715528 |
|  | 15086467 |
|  | 15102771 |
|  | 15147725 |
|  | 15251379 |
|  | 15309723 |
|  | 15385469 |
|  | 15466015 |
|  | 15793858 |
|  | 16002150 |
|  | 16086233 |
|  | 16460340 |
|  | 16461369 |
|  | 16504995 |
|  | 16522370 |
|  | 16523426 |
|  | 16696964 |
|  | 16801669 |
|  | 16804000 |
|  | 16807647 |
|  | 16827395 |
|  | 16949747 |
|  | 17017555 |
|  | 17033197 |
|  | 17042978 |
|  | 17105443 |
|  | 1847510 |
|  | 8228801 |
|  | 8312229 |
|  | 8484757 |
|  | 8499633 |
|  | 9463379 |
|  | 10029084 |
|  | 10049699 |
|  | 10072260 |
|  | 10072487 |
|  | 10072503 |
|  | 10075984 |
|  | 10080539 |
|  | 10213266 |
|  | 10220502 |
|  | 10338525 |
|  | 10349636 |
|  | 10352288 |
|  | 10395689 |
|  | 10415042 |
|  | 10438933 |
|  | 10447769 |
|  | 10452990 |
|  | 10456884 |
|  | 10510089 |
|  | 10512928 |
|  | 10520755 |
|  | 10579119 |
|  | 10580272 |
|  | 10648462 |
|  | 10671193 |
|  | 10680745 |
|  | 10723680 |
|  | 10768980 |
|  | 10770288 |
|  | 10799901 |
|  | 10801266 |
|  | 10806208 |
|  | 10811896 |
|  | 10833484 |
|  | 10849019 |
|  | 10859210 |
|  | 10859330 |
|  | 10896769 |
|  | 10898750 |
|  | 10899905 |
|  | 10946317 |
|  | 10947805 |
|  | 10948132 |
|  | 11009441 |
|  | 11027433 |
|  | 11042159 |
|  | 11053501 |
|  | 11076861 |
|  | 11087155 |
|  | 11091273 |
|  | 11114383 |
|  | 11120869 |
|  | 11145695 |
|  | 11145720 |
|  | 11149562 |
|  | 11154238 |
|  | 11159016 |
|  | 11197687 |
|  | 11217851 |
|  | 11240949 |
|  | 11285204 |
|  | 11290761 |
|  | 11301049 |
|  | 11312256 |
|  | 11337387 |
|  | 11342656 |
|  | 11349033 |
|  | 11353686 |
|  | 11354274 |
|  | 11406467 |
|  | 11438376 |
|  | 11439163 |
|  | 11447117 |
|  | 11465103 |
|  | 11472326 |
|  | 11485976 |
|  | 11489951 |
|  | 11507020 |
|  | 11518689 |
|  | 11544198 |
|  | 11544344 |
|  | 11553589 |
|  | 11592059 |
|  | 11707574 |
|  | 11713466 |
|  | 11714812 |
|  | 11714822 |
|  | 11747355 |
|  | 11751938 |
|  | 11777988 |
|  | 11781183 |
|  | 11781285 |
|  | 11809742 |
|  | 11823481 |
|  | 11823525 |
|  | 11847480 |
|  | 11859129 |
|  | 11869683 |
|  | 11875494 |
|  | 11895333 |
|  | 11895981 |
|  | 11907098 |
|  | 11911827 |
|  | 11920565 |
|  | 11921420 |
|  | 11956064 |
|  | 11970958 |
|  | 11979045 |
|  | 11981816 |
|  | 11984528 |
|  | 11986256 |
|  | 12010973 |
|  | 12016137 |
|  | 12031039 |
|  | 12031983 |
|  | 12055222 |
|  | 12055591 |
|  | 12107190 |
|  | 12147624 |
|  | 12165544 |
|  | 12193690 |
|  | 12213197 |
|  | 12234058 |
|  | 12244307 |
|  | 12391013 |
|  | 12392966 |
|  | 12393933 |
|  | 12406350 |
|  | 12421951 |
|  | 12423305 |
|  | 12443720 |
|  | 12444141 |
|  | 12466851 |
|  | 12477932 |
|  | 12493739 |
|  | 12496393 |
|  | 12505044 |
|  | 12516547 |
|  | 12517950 |
|  | 12517967 |
|  | 12538658 |
|  | 12538682 |
|  | 12540559 |
|  | 12540780 |
|  | 12555667 |
|  | 12591228 |
|  | 12594061 |
|  | 12604029 |
|  | 12644245 |
|  | 12646621 |
|  | 12646644 |
|  | 12648490 |
|  | 12654856 |
|  | 12670447 |
|  | 12672053 |
|  | 12695566 |
|  | 12716449 |
|  | 12734362 |
|  | 12736147 |
|  | 12757625 |
|  | 12759407 |
|  | 12773301 |
|  | 12791309 |
|  | 12791311 |
|  | 12791312 |
|  | 12794151 |
|  | 12813366 |
|  | 12823276 |
|  | 12829028 |
|  | 12842419 |
|  | 12847222 |
|  | 12847269 |
|  | 12855551 |
|  | 12865418 |
|  | 12874231 |
|  | 12902846 |
|  | 12907458 |
|  | 12933825 |
|  | 12938214 |
|  | 12941137 |
|  | 12941477 |
|  | 12960338 |
|  | 12960341 |
|  | 12964046 |
|  | 1350294 |
|  | 13679317 |
|  | 1381512 |
|  | 14500669 |
|  | 14500676 |
|  | 14500760 |
|  | 14511232 |
|  | 14527170 |
|  | 14568011 |
|  | 14568953 |
|  | 14583235 |
|  | 14587096 |
|  | 14597556 |
|  | 14607897 |
|  | 14607938 |
|  | 14625477 |
|  | 14638805 |
|  | 14638808 |
|  | 14659593 |
|  | 14707076 |
|  | 14712288 |
|  | 14734716 |
|  | 14764687 |
|  | 14764735 |
|  | 14975940 |
|  | 14978122 |
|  | 14978125 |
|  | 15016857 |
|  | 15026134 |
|  | 15039316 |
|  | 15039389 |
|  | 15096489 |
|  | 15102048 |
|  | 15102820 |
|  | 15111500 |
|  | 15113757 |
|  | 15116295 |
|  | 15128834 |
|  | 15155614 |
|  | 15158910 |
|  | 1517575 |
|  | 15178562 |
|  | 15187140 |
|  | 15191815 |
|  | 15197230 |
|  | 15218058 |
|  | 15221959 |
|  | 15239105 |
|  | 15240669 |
|  | 15240692 |
|  | 15240714 |
|  | 15240716 |
|  | 15240748 |
|  | 15240754 |
|  | 15251981 |
|  | 15265879 |
|  | 15314695 |
|  | 15319439 |
|  | 15331352 |
|  | 15362035 |
|  | 15364107 |
|  | 15368285 |
|  | 15368446 |
|  | 15381187 |
|  | 15383460 |
|  | 15448706 |
|  | 15465816 |
|  | 15466252 |
|  | 15485815 |
|  | 15491348 |
|  | 15501390 |
|  | 15501460 |
|  | 15501755 |
|  | 15514019 |
|  | 15520859 |
|  | 15522916 |
|  | 15528374 |
|  | 15534372 |
|  | 15538945 |
|  | 15564479 |
|  | 15588938 |
|  | 15593122 |
|  | 15593124 |
|  | 15608148 |
|  | 15611254 |
|  | 15618456 |
|  | 15624697 |
|  | 15634884 |
|  | 15634927 |
|  | 15642955 |
|  | 15652291 |
|  | 15654818 |
|  | 15657087 |
|  | 15661876 |
|  | 15696103 |
|  | 15699124 |
|  | 15728505 |
|  | 15728512 |
|  | 15735429 |
|  | 15749884 |
|  | 15749903 |
|  | 15778345 |
|  | 15784551 |
|  | 15784555 |
|  | 15819696 |
|  | 15825073 |
|  | 15843536 |
|  | 15845473 |
|  | 15879082 |
|  | 15879134 |
|  | 15905543 |
|  | 15919371 |
|  | 15930318 |
|  | 15937545 |
|  | 15944323 |
|  | 15967820 |
|  | 15972681 |
|  | 16002688 |
|  | 16002704 |
|  | 16002724 |
|  | 16005735 |
|  | 16014616 |
|  | 16020505 |
|  | 16041410 |
|  | 16045731 |
|  | 16046470 |
|  | 16081806 |
|  | 16081807 |
|  | 16087867 |
|  | 16113316 |
|  | 16116213 |
|  | 16141072 |
|  | 16141073 |
|  | 16148099 |
|  | 16148101 |
|  | 16148158 |
|  | 16150927 |
|  | 16151533 |
|  | 16166328 |
|  | 16177068 |
|  | 16181351 |
|  | 16184516 |
|  | 16200070 |
|  | 16239532 |
|  | 16256304 |
|  | 16263172 |
|  | 16285949 |
|  | 16299306 |
|  | 16310049 |
|  | 16319715 |
|  | 16339566 |
|  | 16362885 |
|  | 16407368 |
|  | 16428751 |
|  | 16456026 |
|  | 16465591 |
|  | 16467201 |
|  | 16467202 |
|  | 16476050 |
|  | 16476058 |
|  | 16497487 |
|  | 16500554 |
|  | 16513385 |
|  | 16517715 |
|  | 16524889 |
|  | 16600195 |
|  | 16618605 |
|  | 16621988 |
|  | 16622000 |
|  | 16628253 |
|  | 16634801 |
|  | 16636014 |
|  | 16670770 |
|  | 16709607 |
|  | 16714569 |
|  | 16740391 |
|  | 16765924 |
|  | 16769814 |
|  | 16778140 |
|  | 16778988 |
|  | 16782435 |
|  | 16818788 |
|  | 16822852 |
|  | 16861635 |
|  | 16872378 |
|  | 16879997 |
|  | 16885377 |
|  | 16888018 |
|  | 16903779 |
|  | 16914507 |
|  | 16917957 |
|  | 16935932 |
|  | 16936808 |
|  | 16966495 |
|  | 16982822 |
|  | 17002586 |
|  | 17003481 |
|  | 17015698 |
|  | 17015721 |
|  | 17041596 |
|  | 17048272 |
|  | 17057220 |
|  | 17082624 |
|  | 17109466 |
|  | 17111348 |
|  | 17137799 |
|  | 17142729 |
|  | 17148671 |
|  | 17200193 |
|  | 2161559 |
|  | 2531194 |
|  | 7485382 |
|  | 7507352 |
|  | 7525726 |
|  | 7547677 |
|  | 7554464 |
|  | 7594497 |
|  | 7608550 |
|  | 7622047 |
|  | 7696210 |
|  | 7722286 |
|  | 7769128 |
|  | 7783752 |
|  | 7790826 |
|  | 7811281 |
|  | 7930605 |
|  | 7963574 |
|  | 7996051 |
|  | 8035532 |
|  | 8089491 |
|  | 8112615 |
|  | 8116046 |
|  | 8132775 |
|  | 8133052 |
|  | 8168140 |
|  | 8207233 |
|  | 8299735 |
|  | 8313907 |
|  | 8402911 |
|  | 8459215 |
|  | 8605933 |
|  | 8606382 |
|  | 8607899 |
|  | 8622125 |
|  | 8640871 |
|  | 8671667 |
|  | 8691138 |
|  | 8703029 |
|  | 8752931 |
|  | 8770874 |
|  | 8823360 |
|  | 8870704 |
|  | 8871628 |
|  | 8890206 |
|  | 8896500 |
|  | 8977282 |
|  | 8990348 |
|  | 9000540 |
|  | 9036973 |
|  | 9091582 |
|  | 9103224 |
|  | 9120288 |
|  | 9125545 |
|  | 9182682 |
|  | 9184696 |
|  | 9190113 |
|  | 9234781 |
|  | 9292893 |
|  | 9307269 |
|  | 9344502 |
|  | 9366429 |
|  | 9366559 |
|  | 9398398 |
|  | 9498745 |
|  | 9521054 |
|  | 9588906 |
|  | 9610731 |
|  | 9620592 |
|  | 9636366 |
|  | 9665272 |
|  | 9697983 |
|  | 9730890 |
|  | 9759845 |
|  | 9780204 |
|  | 9806746 |
|  | 9811341 |
|  | 9824514 |
|  | 9828225 |
|  | 9831707 |
|  | 9878080 |
|  | 9886396 |
|  | 9891477 |
|  | 9892616 |
|  | 9916075 |
|  | 9916727 |
|  | 9935167 |
|  | 9973435 |
|  | 10064624 |
|  | 10231374 |
|  | 10542212 |
|  | 10714547 |
|  | 10775602 |
|  | 10985253 |
|  | 11044099 |
|  | 11121048 |
|  | 1121048 |
|  | 11441115 |
|  | 11485736 |
|  | 11717514 |
|  | 11737614 |
|  | 11758730 |
|  | 11792123 |
|  | 11805149 |
|  | 11820460 |
|  | 11828010 |
|  | 11829538 |
|  | 11833470 |
|  | 11838849 |
|  | 11857058 |
|  | 11881827 |
|  | 11883743 |
|  | 11896938 |
|  | 11908705 |
|  | 11919157 |
|  | 11920312 |
|  | 11922883 |
|  | 11928840 |
|  | 11929132 |
|  | 11952921 |
|  | 11960393 |
|  | 11964134 |
|  | 11964300 |
|  | 11986609 |
|  | 11991675 |
|  | 11994432 |
|  | 12039409 |
|  | 12042661 |
|  | 12060853 |
|  | 12069178 |
|  | 12069391 |
|  | 12078789 |
|  | 12093676 |
|  | 12095128 |
|  | 12097418 |
|  | 12101075 |
|  | 12115622 |
|  | 12119553 |
|  | 12121678 |
|  | 12145682 |
|  | 12164877 |
|  | 12165551 |
|  | 12170266 |
|  | 12189567 |
|  | 12197884 |
|  | 12202153 |
|  | 12207099 |
|  | 12207325 |
|  | 12207353 |
|  | 12219016 |
|  | 12220553 |
|  | 12223518 |
|  | 12233881 |
|  | 12239181 |
|  | 12370394 |
|  | 12377950 |
|  | 12389080 |
|  | 12393426 |
|  | 12393603 |
|  | 12423294 |
|  | 12438341 |
|  | 12438348 |
|  | 12438424 |
|  | 12444143 |
|  | 12447749 |
|  | 12486603 |
|  | 12495723 |
|  | 12496396 |
|  | 12496489 |
|  | 12525572 |
|  | 12531774 |
|  | 12538698 |
|  | 12548511 |
|  | 12581488 |
|  | 12594817 |
|  | 12595905 |
|  | 12599058 |
|  | 12609526 |
|  | 12615834 |
|  | 12616343 |
|  | 12626585 |
|  | 12640314 |
|  | 12654092 |
|  | 12662377 |
|  | 12663757 |
|  | 12668613 |
|  | 12676754 |
|  | 12700072 |
|  | 12707018 |
|  | 12709682 |
|  | 12714267 |
|  | 12714269 |
|  | 12727335 |
|  | 12730860 |
|  | 12738388 |
|  | 12761561 |
|  | 12778475 |
|  | 12782719 |
|  | 12787424 |
|  | 12788307 |
|  | 12788980 |
|  | 12791310 |
|  | 12791313 |
|  | 12791314 |
|  | 12792880 |
|  | 12802400 |
|  | 12813360 |
|  | 12823999 |
|  | 12824008 |
|  | 12826153 |
|  | 12826369 |
|  | 12847677 |
|  | 12850811 |
|  | 12851716 |
|  | 12861049 |
|  | 12864989 |
|  | 12926133 |
|  | 12934185 |
|  | 12938145 |
|  | 12940515 |
|  | 12944981 |
|  | 12946990 |
|  | 12959221 |
|  | 12967650 |
|  | 13129651 |
|  | 14550257 |
|  | 14567558 |
|  | 14583474 |
|  | 14585195 |
|  | 14592815 |
|  | 14630401 |
|  | 14631168 |
|  | 14638775 |
|  | 14657427 |
|  | 14682393 |
|  | 14697935 |
|  | 14701701 |
|  | 14730222 |
|  | 14746814 |
|  | 14959851 |
|  | 14978075 |
|  | 14994383 |
|  | 14999688 |
|  | 15001471 |
|  | 15016829 |
|  | 15036247 |
|  | 15042002 |
|  | 15057267 |
|  | 15066699 |
|  | 15070757 |
|  | 15072702 |
|  | 15084933 |
|  | 15085177 |
|  | 15086488 |
|  | 15116066 |
|  | 15122773 |
|  | 15122797 |
|  | 15129234 |
|  | 15131577 |
|  | 15144570 |
|  | 15152368 |
|  | 15155645 |
|  | 15179129 |
|  | 15180472 |
|  | 15187110 |
|  | 15188059 |
|  | 15191519 |
|  | 15194284 |
|  | 15200436 |
|  | 15223612 |
|  | 15246967 |
|  | 15271933 |
|  | 15292362 |
|  | 15295621 |
|  | 15306847 |
|  | 15316224 |
|  | 15316663 |
|  | 15325405 |
|  | 15345120 |
|  | 15368517 |
|  | 15381182 |
|  | 15388260 |
|  | 15452323 |
|  | 15462469 |
|  | 15488737 |
|  | 15497451 |
|  | 15527169 |
|  | 15534100 |
|  | 15547296 |
|  | 15551142 |
|  | 15573087 |
|  | 15579372 |
|  | 15596839 |
|  | 15599664 |
|  | 15611648 |
|  | 15622443 |
|  | 15650893 |
|  | 15652446 |
|  | 15659329 |
|  | 15661939 |
|  | 15674653 |
|  | 15682481 |
|  | 15693804 |
|  | 15718915 |
|  | 15728515 |
|  | 15748779 |
|  | 15761493 |
|  | 15780075 |
|  | 15780879 |
|  | 15803357 |
|  | 15810889 |
|  | 15814696 |
|  | 15814743 |
|  | 15815689 |
|  | 15838798 |
|  | 15843082 |
|  | 15845862 |
|  | 15900488 |
|  | 15907845 |
|  | 15927856 |
|  | 15933064 |
|  | 15933743 |
|  | 15940038 |
|  | 15951665 |
|  | 15964392 |
|  | 15978325 |
|  | 15979955 |
|  | 15985167 |
|  | 15990800 |
|  | 16034145 |
|  | 16038043 |
|  | 16043753 |
|  | 16043989 |
|  | 16078336 |
|  | 16084491 |
|  | 16098562 |
|  | 16122836 |
|  | 16162776 |
|  | 16165702 |
|  | 16226734 |
|  | 16256199 |
|  | 16259956 |
|  | 16276011 |
|  | 16305644 |
|  | 16341266 |
|  | 16361810 |
|  | 16364177 |
|  | 16378687 |
|  | 16396704 |
|  | 16413199 |
|  | 16433832 |
|  | 16441483 |
|  | 16460885 |
|  | 16481285 |
|  | 16507146 |
|  | 16551363 |
|  | 16579847 |
|  | 16584395 |
|  | 16611358 |
|  | 16644716 |
|  | 16672419 |
|  | 16690414 |
|  | 16698276 |
|  | 16698426 |
|  | 16699450 |
|  | 16699452 |
|  | 16707559 |
|  | 16720211 |
|  | 16785496 |
|  | 16803619 |
|  | 16809636 |
|  | 16824070 |
|  | 16850164 |
|  | 16861661 |
|  | 16861684 |
|  | 16863661 |
|  | 16889540 |
|  | 16895750 |
|  | 16920714 |
|  | 16924108 |
|  | 16933851 |
|  | 16940525 |
|  | 16945026 |
|  | 16959027 |
|  | 16987066 |
|  | 17003778 |
|  | 17006334 |
|  | 17006724 |
|  | 17015714 |
|  | 17015751 |
|  | 17049666 |
|  | 17052299 |
|  | 17083041 |
|  | 17097497 |
|  | 17133531 |
|  | 17163451 |
|  | 17178344 |
|  | 17188628 |
|  | 2173142 |
|  | 7591871 |
|  | 7695626 |
|  | 7759550 |
|  | 8037735 |
|  | 8248239 |
|  | 8590020 |
|  | 8897595 |
|  | 8898665 |
|  | 8910398 |
|  | 9162098 |
|  | 9312047 |
|  | 9334723 |
|  | 9405662 |
|  | 9637497 |
|  | 9689103 |
|  | 9743208 |
|  | 11196656 |
|  | 11196718 |
|  | 11238636 |
|  | 11477472 |
|  | 12825869 |
|  | 15340161 |
|  | 15837194 |
|  | 16435103 |
|  | 16525502 |
|  | 16690518 |
|  | 16712948 |
|  | 16859503 |
|  | 16872712 |
|  | 16911685 |
|  | 16943796 |
|  | 16978071 |
|  | 17041236 |
|  | 17051272 |
|  | 17092253 |
|  | 17178764 |
|  | 17202341 |
|  | 17204399 |
|  | 17209781 |
|  | 17215862 |
|  | 17225142 |
|  | 17230494 |
|  | 17237407 |
|  | 17237722 |
|  | 17238853 |
|  | 17241871 |
|  | 17261758 |
|  | 17304105 |
|  | 17310845 |
|  | 17314689 |
|  | 17321603 |
|  | 17326156 |
|  | 17331973 |
|  | 17335372 |
|  | 17338374 |
|  | 17380193 |
|  | 17389013 |
|  | 17389235 |
|  | 17393023 |
|  | 17408400 |
|  | 17412916 |
|  | 17413867 |
|  | 17420099 |
|  | 17428673 |
|  | 17431682 |
|  | 17438520 |
|  | 17449476 |
|  | 17460178 |
|  | 17463301 |
|  | 17470530 |
|  | 17490670 |
|  | 17530721 |
|  | 17543146 |
|  | 17544674 |
|  | 17544800 |
|  | 17552041 |
|  | 17559576 |
|  | 17574964 |
|  | 17587429 |
|  | 17587715 |
|  | 17597660 |
|  | 17605296 |
|  | 17617027 |
|  | 17634568 |
|  | 17652887 |
|  | 17665434 |
|  | 17674321 |
|  | 17700165 |
|  | 17716608 |
|  | 7547951 |
|  | 7590988 |
|  | 7749063 |
|  | 8364028 |
|  | 8881045 |
|  | 9043871 |
|  | 9234486 |
|  | 9788463 |
|  | 10754317 |
|  | 10843696 |
|  | 11034365 |
|  | 11035098 |
|  | 11390498 |
|  | 11441071 |
|  | 11489988 |
|  | 11698492 |
|  | 11714756 |
|  | 12218136 |
|  | 12642601 |
|  | 12706496 |
|  | 12799032 |
|  | 15887111 |
|  | 16272349 |
|  | 16275384 |
|  | 16275759 |
|  | 16301610 |
|  | 16314435 |
|  | 16365149 |
|  | 16413748 |
|  | 16488621 |
|  | 16549598 |
|  | 17099102 |
|  | 17116728 |
|  | 17122368 |
|  | 17130251 |
|  | 17157804 |
|  | 17182557 |
|  | 17200198 |
|  | 17202367 |
|  | 17207783 |
|  | 17215490 |
|  | 17228359 |
|  | 17237385 |
|  | 17237391 |
|  | 17238832 |
|  | 17239241 |
|  | 17241399 |
|  | 17295069 |
|  | 17304625 |
|  | 17322422 |
|  | 17353283 |
|  | 17420232 |
|  | 17456802 |
|  | 17464085 |
|  | 17479109 |
|  | 17485448 |
|  | 17485459 |
|  | 17495050 |
|  | 17502388 |
|  | 17505142 |
|  | 17531383 |
|  | 17548634 |
|  | 17574631 |
|  | 17579057 |
|  | 17600128 |
|  | 17618911 |
|  | 17692287 |
|  | 17694059 |
|  | 9432975 |
|  | 9463404 |
|  | 9574553 |
|  | 9892605 |
|  | 17174526 |
|  | 17278885 |
|  | 17336456 |
|  | 17517520 |
|  | 15372997 |
|  | 16111147 |
|  | 16005068 |
|  | 16202237 |

---

|  |  |
| --- | --- |
| Organism | Homo sapiens {Organism urn:agi-taxid:9606} |
|  | Mus musculus {Organism urn:agi-taxid:10090} |
|  | Rattus norvegicus {Organism urn:agi-taxid:10116} |
|  | Mus musculus |
|  | Homo sapiens |
|  | Rattus norvegicus |
|  | Bos taurus |
|  | Danio rerio |

---

|  |  |
| --- | --- |
| Mouse chromosome position | 1 56.89 cM |
|  | 1 69.9 cM |

---

|  |  |
| --- | --- |
| OMIM ID | 124092 |
|  | 614395 |
|  | 609423 |
|  | 180300 |

---

|  |  |
| --- | --- |
| Rat chromosome position | 13q13 |

---

|  |  |
| --- | --- |
| Hugo ID | 5962 |
|  | HGNC:5962 |

---

|  |  |
| --- | --- |
| Human chromosome position | 1q32.1 |
|  | 1q31-q32 |

---

|  |  |
| --- | --- |
| Swiss-Prot Accession | P22301 |
|  | Q6FGW4 |
|  | P22301.1 |
|  | P18893 |
|  | Q3U879 |
|  | P18893.2 |
|  | P29456 |
|  | P29456.1 |
|  | Q0VBJ1 |
|  | Q63263 |
|  | Q6FGS9 |
|  | Q6LBF4 |
|  | Q71UZ1 |
|  | Q9BXR7 |
|  | IL10\_HUMAN |
|  | IL10\_MOUSE |
|  | IL10\_RAT |
|  | Q6FGW4\_HUMAN |
|  | Q0VBJ1\_MOUSE |

---

|  |  |
| --- | --- |
| PIR ID | A38580 |
|  | A34853 |
|  | JN0475 |

---

|  |  |
| --- | --- |
| GenBank ID | NC\_000001 |
|  | NM\_000572 |
|  | NP\_000563 |
|  | NG\_012088 |
|  | AB098711 |
|  | AB261995 |
|  | AB261996 |
|  | AF083251 |
|  | AF247603 |
|  | AF247604 |
|  | AF247605 |
|  | AF247606 |
|  | AF295024 |
|  | AAK19173 |
|  | AF418271 |
|  | AAL06594 |
|  | AL513315 |
|  | CH471100 |
|  | EAW93524 |
|  | DQ217938 |
|  | ABB01008 |
|  | GQ405199 |
|  | ACV30066 |
|  | GQ847742 |
|  | GQ847743 |
|  | JN859070 |
|  | JN859071 |
|  | JN859072 |
|  | JN859073 |
|  | KT291742 |
|  | U06844 |
|  | U16720 |
|  | AAA80104 |
|  | U63015 |
|  | X73536 |
|  | CAA51942 |
|  | X78437 |
|  | CAA55201 |
|  | Z30175 |
|  | CAA82914 |
|  | AF043333 |
|  | AAC03534 |
|  | AY029171 |
|  | AAK38162 |
|  | BC022315 |
|  | BC104252 |
|  | AAI04253 |
|  | BC104253 |
|  | AAI04254 |
|  | CD369014 |
|  | CR541993 |
|  | CAG46790 |
|  | CR542028 |
|  | CAG46825 |
|  | EU751618 |
|  | ACE95684 |
|  | EU751619 |
|  | ACE95685 |
|  | M57627 |
|  | AAA63207 |
|  | P22301 |
|  | NC\_000067 |
|  | NM\_010548 |
|  | NP\_034678 |
|  | AL513351 |
|  | CH466520 |
|  | EDL39722 |
|  | M84340 |
|  | AAA39275 |
|  | AK152344 |
|  | BAE31139 |
|  | BC120612 |
|  | AAI20613 |
|  | BC137844 |
|  | AAI37845 |
|  | M37897 |
|  | AAA39274 |
|  | P18893 |
|  | NC\_005112 |
|  | XM\_006249712 |
|  | XP\_006249774 |
|  | XM\_008769426 |
|  | XP\_008767648 |
|  | NM\_012854 |
|  | NP\_036986 |
|  | AC\_000081 |
|  | AABR07020941 |
|  | AAHX01075050 |
|  | AAHX01075051 |
|  | CH473958 |
|  | EDM09836 |
|  | L02926 |
|  | AAA41425 |
|  | X60675 |
|  | CAA43090 |
|  | P29456 |
|  | AMYH02002554 |
|  | XP\_011507808 |
|  | AAHY01010930 |
|  | XM\_011509506 |
|  | CAH71813 |
|  | AC\_000023 |
|  | AC\_000133 |
|  | NC\_018912 |
|  | AABR06074649 |
|  | ABBA01010192 |
|  | NT\_167186 |
|  | NW\_926794 |
|  | NW\_001838536 |
|  | AC\_000044 |
|  | Q6FGS9 |
|  | Q6FGW4 |
|  | Q6LBF4 |
|  | Q71UZ1 |
|  | Q9BXR7 |
|  | NT\_078297 |
|  | NW\_001030662 |
|  | CAM14013 |
|  | Q0VBJ1 |
|  | Q3U879 |
|  | NW\_047394 |
|  | NW\_001084685 |
|  | NT\_021877 |
|  | NT\_039180 |
|  | NW\_000154 |
|  | 204904 |
|  | 266338 |
|  | 296621 |
|  | 111306698 |
|  | 123789355 |
|  | 123791017 |
|  | 124293 |
|  | 198289 |
|  | 198292 |
|  | 74214592 |
|  | 1041813 |
|  | 119613930 |
|  | 124292 |
|  | 13345058 |
|  | 13774080 |
|  | 14625940 |
|  | 15724398 |
|  | 186271 |
|  | 2905618 |
|  | 49456939 |
|  | 49457009 |
|  | 55665331 |
|  | 74355810 |
|  | 74355812 |
|  | 74709677 |
|  | 74717628 |
|  | 74723161 |
|  | 74724623 |
|  | 74748480 |
|  | 77702090 |
|  | 150439399 |
|  | 122889462 |
|  | 148707775 |
|  | 149058679 |
|  | NT\_086601 |
|  | 1170526 |
|  | 392825 |
|  | AAA19011 |
|  | NC\_007314 |
|  | NM\_174088 |
|  | NP\_776513 |
|  | NW\_001493377 |
|  | P43480 |
|  | U00799 |
|  | 67079379 |
|  | 82194585 |
|  | AAW78362 |
|  | AY887900 |
|  | CR848830 |
|  | NC\_007122 |
|  | NM\_001020785 |
|  | NP\_001018621 |
|  | NW\_001510630 |
|  | Q5EFQ8 |
|  | 67459915 |
|  | 106880414 |
|  | 126003251 |
|  | 67459916 |
|  | 553957 |
|  | Dr.135567 |
|  | ZDB-GENE-051111-1 |
|  | dre:553957 |

---

|  |  |
| --- | --- |
| Swiss-Prot ID | IL10\_HUMAN |
|  | IL10\_MOUSE |
|  | IL10\_RAT |
|  | Q3U879\_MOUSE |
|  | Q0VBJ1\_MOUSE |
|  | Q6FGW4\_HUMAN |

---

|  |  |
| --- | --- |
| Cell Localization | Secreted |
|  | Extracellular |

---

|  |  |
| --- | --- |
| Ensembl ID | ENSG00000136634 |
|  | ENSP00000412237.1 |
|  | ENST00000423557.1 |
|  | ENSMUSG00000016529 |
|  | ENSMUSP00000016673.5 |
|  | ENSMUST00000016673.5 |
|  | ENSRNOG00000004647 |
|  | ENSRNOP00000006246.2 |
|  | ENSRNOT00000006246.5 |
|  | ENSMUST00000016673 |
|  | ENSMUSP00000016673 |
|  | ENSP00000412237 |
|  | ENSRNOP00000006246 |
|  | ENST00000423557 |
|  | ENSRNOT00000006246 |

---

|  |  |
| --- | --- |
| MGI ID | MGI:96537 |
|  | 96537 |

---

|  |  |
| --- | --- |
| RGD ID | 2886 |

---

|  |  |
| --- | --- |
| Unigene ID | Rn.9868 |
|  | Hs.193717 |
|  | Mm.874 |
|  | Bt.4723 |
|  | Dr.135567 |

---

|  |  |
| --- | --- |
| Homologene ID | 478 |

---

|  |  |
| --- | --- |
| Shape | Rhomb |

---

|  |  |
| --- | --- |
| IPI ID | IPI00026102 |
|  | IPI00654087 |
|  | IPI00326111 |
|  | IPI00115602 |

---

|  |  |
| --- | --- |
| Microarray ID | 100319\_at |
|  | AFFX-MurIL10\_at |

---

|  |  |
| --- | --- |
| Homo sapiens Chromosome position | 1q31-q32 |

---

|  |  |
| --- | --- |
| Ensembl Transcript ID | ENST00000367099 |

---

|  |  |
| --- | --- |
| FunctionalClass | cytokine |
|  | interleukin-10 receptor binding |
|  | protein binding |
|  | growth factor |

---

|  |  |
| --- | --- |
| Bos taurus Chromosome position | 16 |

---

|  |  |
| --- | --- |
| Mus musculus Chromosome position | 1 69.9 cM |

---

|  |  |
| --- | --- |
| KEGG pathway | Cytokine-cytokine receptor interaction |
|  | Jak-STAT signaling pathway |
|  | T cell receptor signaling pathway |

---

|  |  |
| --- | --- |
| Rattus norvegicus Chromosome position | 13q13 |

---

|  |  |
| --- | --- |
| Ensembl Gene ID | ENSG00000136634 |

---
